# Functional analysis of a pathogenesis-related thaumatin-like protein gene *TaLr35PR5* from wheat induced by leaf rust fungus

**DOI:** 10.1186/s12870-018-1297-2

**Published:** 2018-05-04

**Authors:** Jiarui Zhang, Fei Wang, Fang Liang, Yanjun Zhang, Lisong Ma, Haiyan Wang, Daqun Liu

**Affiliations:** 1 Center of Plant Disease and Plant Pests of Hebei Province, College of Plant Protection, Hebei Agricultural University, Baoding, 071001 China; 2 Division of Plant Science, Research School of Biology, Australian National University, ACT, Acton, 2601 Australia; 3 Graduate School of Chinese Academy of Agricultural Sciences, Beijing, 100081 China

**Keywords:** Localization, Silencing, TaLr35PR5, Thaumatin-like protein, Wheat

## Abstract

**Background:**

Plants have evolved multifaceted defence mechanisms to resist pathogen infection. Production of the pathogenesis-related (PR) proteins in response to pathogen attack has been implicated in plant disease resistance specialized in systemic-acquired resistance (SAR). Our earlier studies have reported that a full length *TaLr35PR5* gene, encoding a protein exhibiting amino acid and structural similarity to a sweet protein thaumatin, was isolated from wheat near-isogenic line TcLr35. The present study aims to understand the function of *TaLr35PR5* gene in *Lr35*-mediated adult resistance to *Puccinia triticina*.

**Results:**

We determined that the TaLr35PR5 protein contained a functional secretion peptide by utilizing the yeast signal sequence trap system. Using a heterologous expression assay on onion epidermal cells we found that TaLr35PR5 protein was secreted into the apoplast of onion cell. Expression of *TaLr35PR5* was significantly reduced in BSMV-induced gene silenced wheat plants, and pathology test on these silenced plants revealed that *Lr35-*mediated resistance phenotype was obviously altered, indicating that *Lr35*-mediated resistance was compromised.

**Conclusions:**

All these findings strongly suggest that *TaLr35PR5* is involved in *Lr35*-mediated adult wheat defense in response to leaf rust attack.

**Electronic supplementary material:**

The online version of this article (10.1186/s12870-018-1297-2) contains supplementary material, which is available to authorized users.

## Background

Plants have developed sophisticated mechanisms to combat pathogen infection. One of the acquired modes in response to pathogen attack is the production of the pathogenesis-related (PR) proteins, which play key roles in plant disease-resistance responses, specialized in systemic-acquired resistance (SAR) [1]. Seventeen different groups of PRs have been identified so far, and one of these important groups is PR-5 that shares homology with a sweet taste protein, named as thaumatin, from West African rain forest shrub *Thaumatococcus danielli* [2]. Therefore, this group is also defined as thaumamatin like proteins (TLPs). *TLPs* are able to rapidly accumulate to high levels in response to biotic or abiotic stress and exhibit antifungal activity in various plant species [3]. Cao et al. reported that most TLP members in *Arabidopsis*, *Oryza*, *Populus*, *Zea*, *Physcomitrella* and *Chlamydomonas* respond to some biotic or abiotic stresses and some resistance genes function together with *TLPs* based on the genome-wide and functional network analysis in these species [4]. It has been documented that over-expression of *TLPs* is able to induce antifungal activity in different transgenic plants [3]. Transgenic potato plants overexpressing a tobacco *TPL* gene display enhanced fungal resistance [5]. Similarly, potato plants overexpressing *Camellia sinensis TLP* gene (*CsTLP*) confer resistance against two fungal pathogens that differ in life style [6]. Transgenic creeping bentgrass overexpressing a rice thaumatin-like protein (TLPD34) gene exhibits improved resistance to dollar-spot in the field tests [7]. In addition to antifungal activities, many studies showed that *TLPs* exhibit enzymatic activities and play roles in developmental processes including glucanase activity [8], xylanase inhibitor [9], anti-pest activities [10, 11], antifreeze activities [12], seed germination [13] and senescence [14]. Singh et al. examined the patio-temporal expression profiling of thaumatin-like protein during compatible and incompatible interaction, and attributed the differences to the presence of seedling leaf rust resistance *Lr28* gene, which facilitate prevention of leaf rust infection in resistant wheat plants [15].

Majority of TLPs contain 16 cysteine residues that are able to form eight disulfide bonds. Three-dimensional structure of TLP revealed that it consists of three domains and these disulfide bonds are distributed in these three domains, and an interdomain cleft is observed between domains I and II [16, 17]. This structure is able to stabilize protein, which allows the protein to resist to pH, proteases and heat-induced denaturation [18]. It is well accepted that the highly acidic interdomain cleft of the TLPs strongly correlates with their antifungal and β-glucanase activities [19–21]. However, recent studies found that the PcOSM1 from *Phytophthora*-resistant wild pepper has significantly reduced antifungal activity despite of the presence of a strong acidic cleft. This finding suggests that the presence of acidic cleft alone may not be sufficient for the antifungal activity, indicating other structural features like domain I or/and III is likely required for the activity [22, 23].

Secretion of proteins from ribosomes to outside of the cell requires the effective translocation of the protein across the endoplasmic reticulum (ER). This process is mediated by secretory signal peptide of the protein [24]. Signal peptide is located at N-terminus of proteins with the ranges of between 15 and 30 amino acids and is usually cleaved off during translocation across the endoplasmic reticulum membrane [25]. Since the principles of protein translocation mechanism are evolutionarily conserved in prokaryote and eukaryote [26], it is evident that secretion signal is functional in cross-host. Recent years, yeast signal sequence trap (YSST) has been successfully developed to identify secreted proteins originally from animals, plants and fungal pathogens [27]. In yeast the secreted invertase encoded by *SUC2* is a key enzyme to catalyse sucrose to generate glucose and fructose to supply carbon sources for yeast growth. YSST utilizes a vector named as pSUC2T7M13ORI with a *SUC2* gene lacking the start codon and signal peptide and the host *SUC2*-deficient strain of *Saccharomyces cerevisiae* that is unable to secrete invertase resulting in the grow on sucrose selection medium [28]. YSST has been effectively applied to identify secreted proteins from plants and plant pathogens including such as *P. infestans* [29] and *Colletotrichum graminicola* [27].

Leaf rust, caused by *Puccinia triticina*, is one of the most destructive diseases in the major wheat-growing regions [30]. It can reduce the quality of harvested grain and cause significant yield losses. Development of genetic resistance to rust has been approved as the most efficient, cost-effective, and environment-friendly approach to prevent the losses due to rust epidemics [31]. To date, 76 leaf rust resistance genes, named as *Lr* genes, have been identified and confer broaden resistance to seedling and adult plants [32]. One of adult plant resistance genes *Lr35* expressing from two-leaf stage confers durable and effective resistance over a long period of time against diverse pathotypes of the fungus [33]. In our previous study, a full-length *PR5* gene designated as *TaLr35PR5* has been cloned from wheat near-isogenic lines TcLr35 in response to the infection of *P. triticina* pathotype PHNT. *TaLr35PR5* gene encodes a 171-amino acid protein that exhibits high homology with a sweet protein thaumatin and displayed early and high expression in incompatible interaction compared with compatible interaction [34]. In this study, we identify and clone the signal peptide of TaLr35PR5 protein, and examine the function of the signal peptide. We also study the subcellular localization of TaLr35PR5 protein using heterologous expression system. In addition, we assess the rust disease development on the BSMV-induced *TaLr35PR5* silenced *Lr35* wheat plants.

## Methods

### Plant materials and leaf rust isolate

Wheat cultivar Thatcher, which is susceptible to all tested *P. triticiina* pathotypes, and TcLr35 (Tc*6/RL6082), which is a near-isogenic line of the spring wheat line containing *Lr35* gene, were used in this study. These two wheat lines were originally requested from the Cereal Disease Lab of USDA located at University of Minnesota (https://www.ars.usda.gov/midwest-area/stpaul/cereal-disease-lab/) and were preserved in the Laboratory of Leaf rust, Hebei Agricultural University. Plant growth condition and leaf rust pathotype used in this study were described previously [35]. Briefly, the plants were grown to adult stage in the greenhouse at 20 °C, 16 h days, 350 lx light and 80% relative humidity. The single-spore culture of *P. triticina* pathotype 07-10-426-1 (PHNT) was used in this study, which causes an incompatible (TcLr35- PHNT, type 1) on TcLr35 plant and compatible interaction (Thatcher-PHNT, type 4) on Thatcher plant according to Roelfs’ standard [36].

### Bioinformatics analysis of TaLr35PR5

ExPASyProtparam and Protscale were employed to determine the amino acids compositions, physical and chemical properties and the hydrophobicity or hydrophilicity. Psort Prediction (http://psort.hgc.jp/form.html)and TargetP (http://www.cbs.dtu.dk/services/TargetP/) were used to analyze the subcellular localization. Prediction of signal peptide was performed using SignalP 4.0(http://www.cbs.dtu.dk/services/SignalP-4.0/).

### RNA extraction and cDNA synthesis

Total RNA extraction and cDNA synthesis were described previously [37]. Briefly, total RNA was extracted from the fully expended wheat leaf 12 to 14 days after *P. triticina* pathotype 07-10-426-1 inoculation according to the protocol of the TaKaRa MiniBEST Universal RNA Extraction Kit (TaKaRa, Japan). DNA was removed with amplification-grade DNAse I (TaKaRa, Japan) and cDNA was synthesized using the SuperScript II reverse transcriptase, oligo(dT)12–18, and RNAse OUT (Invitrogen). Synthesized complementary DNA (cDNA) was treated with RNAse H and used as the template for quantitative reverse transcription PCR (qRT-PCR).

### Vector construction

To generate the construct used for the yeast signal trap system experiments, the signal peptide fragment of *TaLr35PR5* gene was obtained via PCR. The amplification was performed with primer pair TaLr35PR5-SP-F:5-CGGAATTCATGGCGACCTCCGCGGTGCTC-3/TaLr35PR5-SP-R: 5-AATCTCGAGGGTGGCCGCGCTGGCACCGGC-3 using wheat cDNA as template. The obtained product, carrying *Eco*RI and *Xho*I restriction sites, was cloned into the vector pSUC2T7M13ORI (pSUC2) digested with the same restriction enzymes [27].

For subcellular localization study, full length of *TaLr35PR5* with native signal peptide flanked with *Xba*I and *Kpn*I restriction sites was amplified using primer set TaLr35PR5-Y-: 5-GCTCTAGAATGGCGACCTCCGCGGTGCTC-3 and TaLr35PR5-Y-R: 5-GGGGTACCTCATGGACAGAAGGTGATC-3 with wheat cDNA as template. The obtained product was cloned into the vector pCamA-GFP digested with *Xba*I and *Kpn*I. The resulting construct pCamA::*TaLr35PR5-GFP* encodes the TaLr35PR5 proteins that carry an C-terminal GFP tag.

To generate the constructs used for BSMV-induced gene silencing experiments, two *TaLr35PR5* DNA fragments were obtained via PCR. The amplifications were performed with with gene specific primer pair V-PR5-1F: 5-ATATTAATTAAGCGACCTCCGCGGTGCTCTT-3/V-PR5-1R:5-TATGCGGCCGCGTCGCCGGTCTGGCAGC-3 and V-PR5-2F:5-ATATTAATTAAGCCAGCTGTCCTGCTCCCTC-3/V-PR5-2R:5-TATGCGGCCGCCATGGACAGAAGGTGATCTGGT-3 using pCamA::*TaLr35PR5-GFP* as template, respectively. The obtained fragments were named as V1 and V2, respectively. Subsequently, the V1 and V2 DNA fragments were digested with *Pac*I and *Not*I restriction enzymes and cloned to the γ vector digested with the same restriction enzymes to generate the p γ::BMSV-V1 and p γ::BMSV-V2, respectively [38].

### Yeast signal sequence trap system

Yeast transformation was performed according to the protocol listed in the Yeastmaker™ Yeast Transformation System 2 (Clontech, USA). The invertase negative yeast strain YTK12 that was kindly provided by Professor Xiaofeng Dai from Chinese Agriculture Academy was transformed with 20 ng of the pSUC2::*TaLr35PR5*. pSUC2-Mg87 and pSUC2-Ps87, kindly provided as a gift by Dr. Xiaodong Wang from Hebei Agricultural University, were separately transformed as the negative and positive controls. After transformation, yeast was plated on CMD-W (minus Trp) plates (0.67% yeast N base without amino acids, 0.075% W dropout supplement, 2% sucrose, 0.1% glucose, and 2% agar). Transformed colonies were transferred to fresh CMD-W plates and incubated at 30 °C for 3 days.

For the invertase secretion assay, transformed colonies were replica plated on CMD-W plates and YPRAA plates (1% yeast extract, 2% peptone, 2% raffinose, and 2 μg/mL antimicyn A) containing raffinose and lacking glucose. After 3 days incubation at 30 °C, the plates were checked for growth and photographed. All PCR primers were purchased from Sangon Biotech (Shanghai) Co., Ltd. and sequences of all plasmids were confirmed by sequence analysis.

### Particle bombardment-mediated transient transformation of onion epidermal cells

The generated vector pCamA::*TaLr35PR5*-GFP and positive control pCamA::*GFP* were transformed into onion epidermal cells by particle bombardment at a helium pressure of 7.6 MPa (1100 psi) using the PDS-1000/He system (Bio-Rad, Hercules, CA). After that, the transformed onions were incubated at 25 °C for 1 day in dark. For plasmolysis assay, the transformed onion cells were treated with 40% sucrose for 10 min. Confocal microscopy was performed with Olympus Fluoview FV10i (Olympus, Japan). Excitation of the GFP was done at 488 nm and emission was captured with a 505-530 nm pass filter. The experiments were repeated at least twice with consistent results.

### Quantitative RT-PCR analysis

qRT-PCR was performed with the iCycler IQ real-time detection system (Bio-Rad, Amsterdam, Netherlands). Expression of *TaLr35PR5* was investigated using qPR5-F: 5’-GGGATCCATGGCGACCTCCGCGGTGCTC-3′ and qPR5-R: 5’-CCAAGCTTTCATGGACAGAAGGTGATCTGGTC-3′. Expression of wheat glyceraldehyde-3-phosphate dehydrogenase (GAPDH, GenBank accession No. AF251217) gene was used to calibrate the expression level of the query genes, as previously described [37]. Quantification of the target gene was assessed by relative standard curves. The 2^−ΔΔ^*Ct* [39] method was employed to quantify the relative gene expression. The statistical significance of differences was calculated using GraphPad Prism 6 (GraphPad Software, Inc., USA) with One-way ANOVA followed by the Turkey post-test to obtain the *P*-value. Data were shown as mean ± SEM of three biological replicates from one representative experiment. Significant differences between treatments and controls were represented by three asterisks (*P* < 0.001).

### Barley stripe mosaic virus*-*mediated gene silencing in wheat

The plasmids corresponding to the three RNA fragments, α, β, and γ of BSMV were prepared according to the previously described method with few modifications [40]. Briefly, to generate these three transcripts in vitro, three plasmids were linearized with restriction enzymes *Mlu*I (α component), SpeI (β component) or *Bss*HII (γ component), respectively. In vitro transcription was performed using the mMESSAGE mMACHINE® Kit High Yield Capped RNA Transcription Kit (Ambion) with linearized plasmid as template according to the manufacturers protocol. The concentration of transcribed RNA was adjusted to 1 μg/μl and 10 μl of each of three transcripts were combined and mixed with 90 μl DEPC water and 120 μl of GKP buffer (1 g bentonite, 1 g diatomite, 0.375 g glycine, and 0.523 g K_2_HPO_4_, mixed with 100 μl DEPC water).

BSMV inoculation was performed following the method described previously [40]. Briefly, 240 μl mixture was applied to the fully expended leaf of 7 or 8-leave stage old TcLr35 wheat plants by rubbing. To improve virus infection, the inoculated plants were sprayed with DEPC water without forming droplets on the plants and covered with a plastic bag for 24 h at 23 °C and then transferred them to greenhouse with controlled settings. The pγ:PDS harbouring a 185 bp-fragment from the barley *phytoene desaturase* (*PDS*) gene was used a positive control and pγ::00 was used as a negative control. Non-inoculated TcLr35 and Thatcher wheat plants were used as controls as well.

### Leaf rust inoculation

Ten days after BSMV inoculation, leaf rust urediospores were applied with a brush to the surface of the primary leaves of TcLr35 and susceptible wheat cultivar Thatcher. Sterile water was inoculated to wheat leaves as control. After inoculation, the plants were kept at 100% relative humidity in the dark for 24 h at 20 °C, and then moved to the greenhouse. Twelve to 14 days after inoculation, the disease development was recorded and photographed. Meanwhile, samples were collected for the purpose of histological observations of hyphae development and extraction of total RNA for qPCR analysis to check the expression of *TaLr35PR5*.

### Histological observation of fungal growth

Harvested samples were decolorized as described previously [41]. Leaf segments cut from the transparent wheat leaves were examined using an Olympus BX-51 microscope (Olympus Corp., Tokyo, Japan). For each treatment, at least 60 different infection sites were examined on each of five randomly selected leaf segments.

## Results

### Bioinformatics analysis of TaLr35PR5 protein

**Fig. 1:**
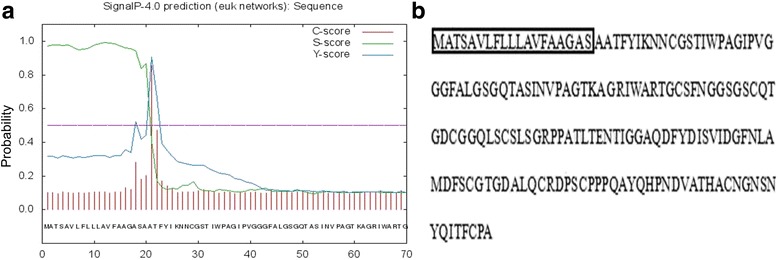
The graphical output of SignalP4.0 predictions (**a**) and the predicted amino acids sequences and signal peptide sequences (highlighted with rectangle) of TaLr35PR5 protein (**b**)

The *TaLr35PR5* gene was predicted to encode a 17.3 kDa protein corresponding to 171 amino acids and containing 10 cysteines (Fig. 1b). Protscale analysis revealed the hydrophilic, acidic and highly stable properties of TaLr35PR5. Psort and TargetP1.1 analyses showed that TaLr35PR5 protein was predicted to be secreted outside of plant cell with the likelihood of 77.8% and no mitochondria localization signal present in the protein. In addition, we employed Signal IP4.0 [42] to accurately predict the presence and cleavage sites of signal peptide. Figure 1 showed that Y-score peaked at the 21st amino acid, where C-score peaked coincide with the transition from a high to a low of S-score, indicating that a robust signal peptide is present in TaLr35PR5 protein and the cleavage site is at position of the maximal Y-score after the 21st amino acid (Fig. 1).

### Functional analysis of signal peptide

**Fig. 2:**
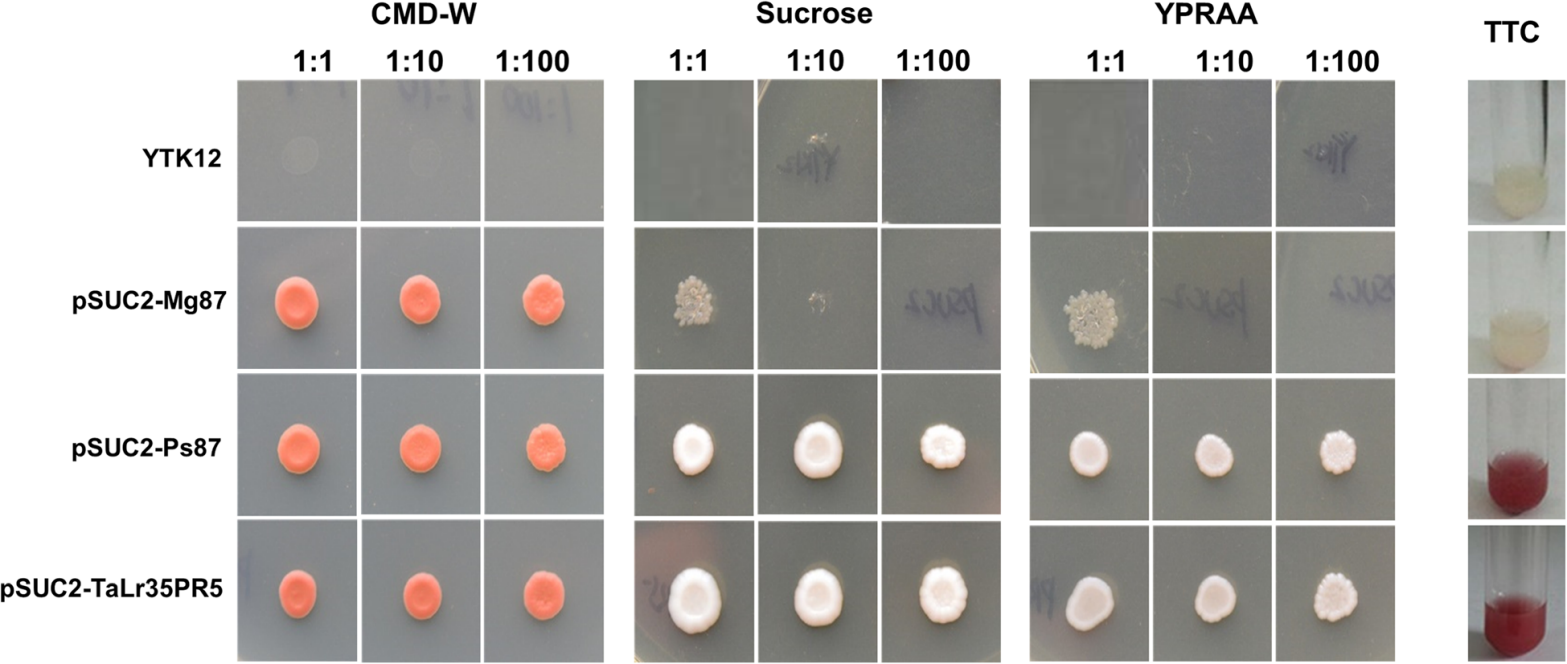
Yeast signal sequence trap assay of the predicted signal peptide of TaLr35PR5 protein. The predicted signal peptide sequences plus the following two amino acids (1–23) of TaLr35PR5, the positive control pSUC2:: Ps87 and negative control pSUC2::Mg87 were used for the assay. CMD-W (minus Trp) plates were used to select yeast strain YTK12 carrying the pSUC2 vector. Sucrose and YPRAA media were used to indicate invertase secretion. An enzymatic activity test based on conversion of the dye 2, 3, 5-triphenyltetrazolium chloride (TTC) to the insoluble red colored triphenylformazan was used to confirm invertase secretion

To examine the secretary function of the signal peptide of TaLr35PR5 protein, we utilized the yeast signal sequence trap system [28]. Specific primers TaLr35PR5-SP-F/TaLr35PR5-SP-R were designed to clone the predicted signal peptide sequences of *TaLr35PR5* gene. Cloning and validation of the obtained construct pSUC2::*TaLr35PR5-SP* were showed in Additional file 1: Figure S1 and Additional file 2: Figure S2. The resulting construct pSUC2::*TaLr35PR5-SP,* negative control pSUC2::Mg87 and positive control pSUC2::Ps87 were separately transformed to yeast strain YKT12 which is deficient in the yeast intervase gene. As shown in Fig. 2 the pSUC2::*TaLr35PR5-SP* or the positive control pSUC2::Ps87 enabled the invertase-deficient yeast strain YKT12 to grow on sucrose and YPRAA medium. As expected, both yeasts carrying the individual construct secreted invertase into the medium which was evidenced by catalyzing conversion of the dye 2, 3, 5-triphenyltetrazolium chloride (TTC) to the insoluble red colored triphenylformazan. Conversely, the negative controls (yeast strain YTK12 and YTK12 carrying the pSUC2::Mg87) were not able to grow on YPRAA plates, and TTC-treated cultures remained colorless (Fig. 2). These results demonstrated that TaLr35PR5 carried a functional secretory signal peptide.

### Localization of TaLr35PR5 protein in plant cell

**Fig. 3:**
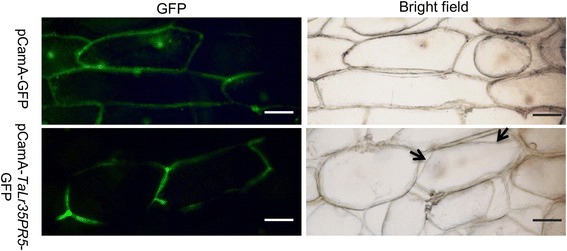
TaLr35PR5 is secreted and localizes in the apoplastic space of onion epidermal cells. Epidermal cells of onion transiently expressing GFP alone or TaLr35PR5-GFP were observed using confocal microscopy at 16 h post particle bombardment-mediated transient expression after plasmolysis. Scale bars = 50 μm

To further assess the secretion and localization of TaLr35PR5 protein in vivo, we generated the fusion construct pCamA::*TaLr35PR5-GFP* in which *TaLr35PR5* was fused to GFP at its C-terminus for bombardment-mediated transient assay on onion epidermal cells. Validation of this construct was showed in Additional file 3: Figure S3 and Additional file 4: Figure S4. The empty vector pCamA::*GFP* was used as a negative control. Confocal microscopic observation showed that the control GFP protein localized both in cytoplasm and nucleus (Fig. 3). In contrast, TaLr35PR5-GFP signals were clearly visualized in the apoplastic space (AP) as evidenced by the plasmolysis (Fig. 3) and no GFP signals were observed in the nucleus. Based on these observations, we concluded that TaLr35PR5 was secreted outside of plant cell, which was consistent with the predicted subcellular localization.

### Disease development on *TaLr35PR5* silenced TcLr35 wheat plants

**Fig. 4:**
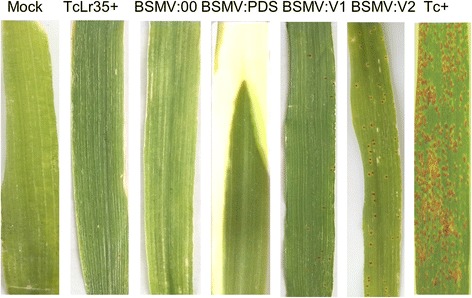
Effect of BSMV-induced *TaLr35PR5* silencing on disease development in wheat. Seventh leaves of 7-leave stage old TcLr35 wheat plants were inoculated with viral vectors as a transcripts mixture containing α, β, and γ carrying derivatives of recombinant γ vector either no insert (BSMV:00) or containing a *PDS* gene (BSMV::*PDS*) or two individual *TaLr35PR5* silencing fragments (BMSV::V1 or BSMV:: V2). BSMV::00 and BSMV::PDS were used as negative and positive control, respectively. Plants were spray-inoculated with *Pt* uridiniospores 10 days after virus or buffer inoculations. Disease developments were monitored 14-days after dridiniospores inoculation and representative leaves were photographed

To assess the role of *TaLr35PR5* in *Lr35*-meidated resistance, BSMV-induced gene silencing was employed to knockdown the expression of *TaLr35PR5* in TcLr35 plants. Validation of silencing constructs: p γ::V1 and p γ::V2 was showed in Additional file 5: Figure S5 and Additional file 6: Figure S6. A silencing construct targeting the wheat PDS was included to monitor the silencing efficiency and progression (silencing of PDS results in a photo bleached leaf phenotype). An empty pγ construct was used as a negative control. As expected, 7-10 days after inoculation, all inoculated plants displayed typical systemic mosaic viral symptoms of BSMV and additional visible photobleached symptoms in the *PDS* silenced plants confirming the onset of gene silencing (Fig. 4).

**Fig. 5:**
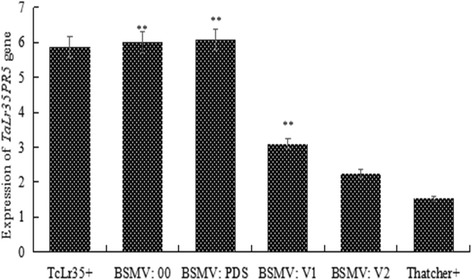
Quantitative PCR analysis showing *TaLr35PR5* expression in TcLr35 wheat plants inoculated with individual BSMV constructs and leaf rust pathogens, in wild-type (Mock) without BSMV and leaf rust inoculations, and in susceptible Thatcher wheat plant inoculated only with leaf rust pathogens. *TaLr35PR5* gene expression levels are normalized to that of GAPDH. Values are means of triplicate reactions of three independent biological samples. Significant differences are represented by asterisks and error bars represent the standard deviation

Ten days after BSMV inoculation, all infected plants were challenged with *P. triticina* urediniospores and the disease development was examined 14-day after inoculation. Figure 4 showed that the TcLr35 plants inoculated with BSMV::00 and BSMV::*PDS* exhibited incompatible phenotype with reaction type 1 based on a modified Cobb scale and the same phenotype was observed in TcLr35 plants without BSMV inoculation, indicating that inoculation of BSMV had no impact on *Lr35*-meidated resistance. However, the TcLr35 plants inoculated with BSMV::V1 displayed a limited numbers of lesions with production of leaf rust uredinia corresponding to the reaction type 2. Surprisingly, the TcLr35 plants inoculated with BSMV::V2 exhibited more sever disease developments as evidenced by producing more leaf rust uredinia, which was scored as reaction type 3 (Fig. 4). qPCR confirmed that *TaLr35PR5* expression levels was 50% and 80 reduced upon inoculation with BSMV::V1 and BSMV::V2 compared with the wildtype TcLr35 plants without any treatments, indicating that both silencing constructs differed in the silencing efficiency and expression of *TaLr35PR5* was highly reduced in BSMV::V2 inoculated plants compared to BSMV::V1 inoculated plants (Fig. 5). As expected, the expression of *TaLr35PR5* in TcLr35 or TcLr35 inoculated with BSMV::00 or BSMV::PDS was significantly increased compared with wildtype TcLr35 plants without any treatments (Fig. 5), which was in agreement with our previous findings [35]. Taken together, these findings strongly indicated that *TaLr35PR5* was required for *Lr35*-mediated resistance against leaf rust fungus.

### Histological observation of *TaLr35PR5* silenced leaves infected with *Puccinia triticina*

**Fig. 6:**
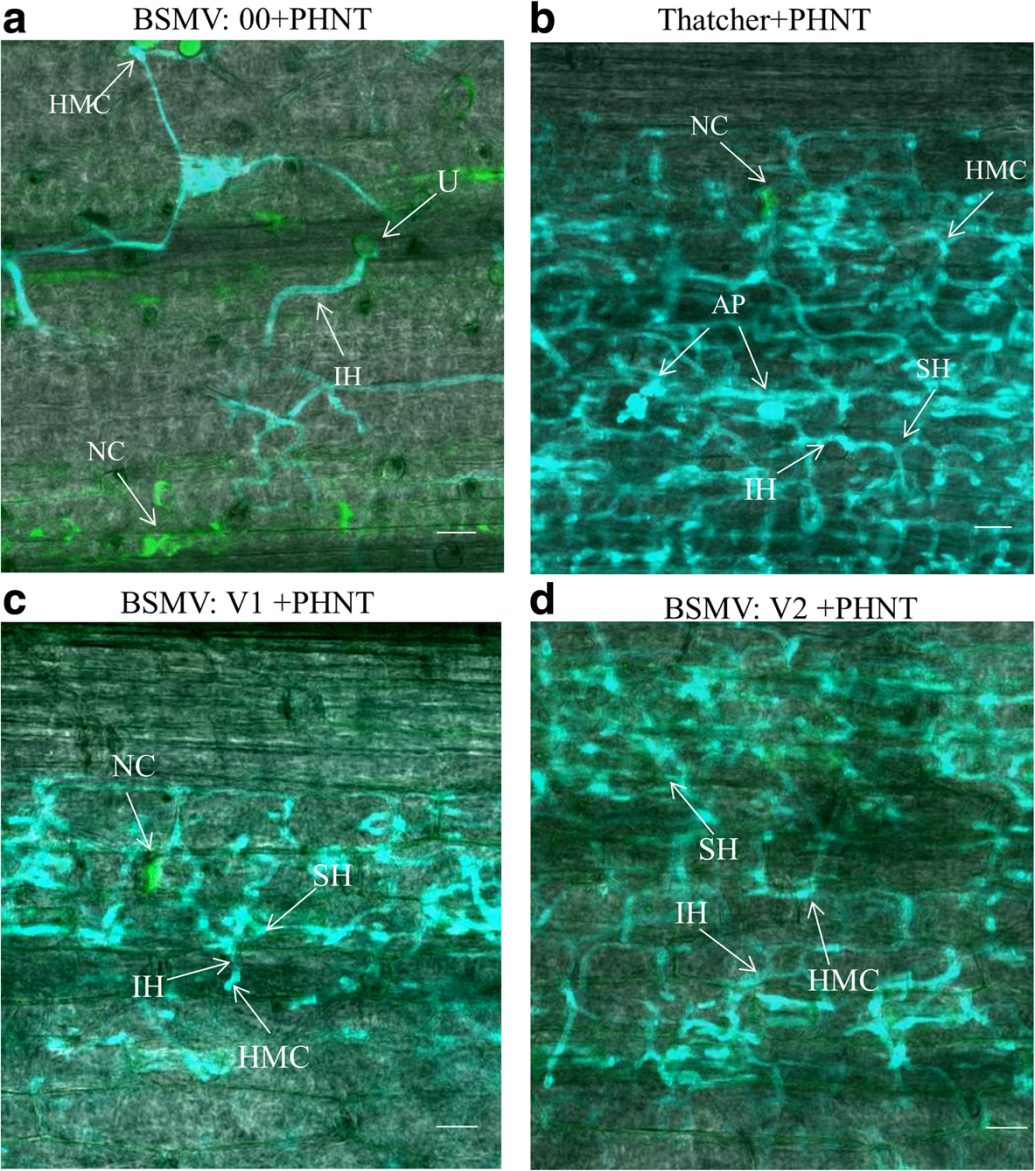
Histological observation of hypha development and host cell death in wheat leaves treated with recombinant Barley stripe mosaic virus (BSMV) viruses and infected with PHNT. **a**. TcLr35 plants inoculated with BSMV::00 and PHNT. **b**. Susceptible control plants (Thatcher) without BSMV treatment after PHNT infection. **c**. Silenced TcLr35 plants inoculated with BSMV::V1 and PHNT. **d**. Silenced TcLr35 plants inoculated with BSMV::V2 and PHNT. IH: infection hypha; HMC: haustorial mother cell; SH: second hypha; NC: necrotic cell; U: Urediospore; AP: appresorium

To confirm the effect of silenced *TaLr35PR5* on the development of fungal structures inside TcLr35 host cells, we visualized the leaves of BSMV-inoculated *Lr35* plants and non-inoculated susceptible plants (Thatcher) inoculated with *Pt*. Confocal microscopic observation showed that the growth of fungus was significantly restricted with mycelia being confined to the first few mesophyll cells in close vicinity of the substomatal cavity and few haustoria formed, but many necrotic cells were observed in TcLr35 plants inoculated with BSMV::00 (Fig. 6a). Surprisingly, the silenced TcLr35 plants inoculated with BSMV::V2 in which *Pt* infection was greatly increased, indicating decreased arrest of mycelial growth as evidenced by many secondary hyphal growths with increasing formation of haustoria (Fig. 6d). In the silenced TcLr35 plants inoculated with BSMV::V1 the growth of fungus remained confined and restricted in which restricted intercellular secondary hyphal growth with few additional haustoria were formed and visible necrotic cells were observed (Fig. 6c). As we expected, the susceptible control plants (Thatcher) without BSMV treatment after *Pt* infection showed extensive mycelia growth, colonization of mesophyll cells, a prolific formation of haustoria, but limited necrotic cells (Fig. 6b). We therefore concluded that silencing *TaLr35PR5* compromised *Lr*35-mediated resistance. These findings correlated well with the observations of rust disease developments implying that enhanced rust growth was observed in silenced TcLr35 plants.

## Discussion

Plants mainly rely on the physical barriers and induced innate immunity systems to combat pathogen infections [43]. One of the acquired resistances in response to pathogen attack is the production of PR proteins. *PR5*, encoding a protein sharing homology with a sweet protein thaumatin and also being defined as TLPs, was first isolated from *Thaumatococcus daniellii* [2]. Here, we present the characterization and functional analysis of the TaLr35PR5. Using heterologous expression system and BSMV-induced gene silencing approach, we demonstrated that TaLr35PR5 is secreted into the apoplast of plant cell and *Lr35* resistance phenotype is compromised when expression of *TaLr35PR5* is significantly reduced. This is the first example of study on *TLPs* gene involving in *Lr35*-mediated resistance against *P. triticina*.

Previous study on *TaPR5* that was isolated from wheat leaves (cv. Suwon 11) infected by the stripe rust pathotype CY23 showed that TaPR5 share a significant sequence similarity with PR5 and TLPs from barley and other plants [44]. Comparisons of predicted amino acid sequences between TaLr35PR5 and TaPR5 uncovered that both protein share 92% similarity and 10 conserved cysteines (Additional file 7: Figure S7). Because of the predicted signal peptide at the N-terminus and the acidic property, it is suggested that TaLr35PR5 protein may be secreted extracellularly. Using yeast signal sequence trap system and particle bombardment-mediated transient assay, the function of signal peptide was confirmed and the extracellular localization was determined. The TaLr35PR5–GFP fusion protein was imaged in the apoplast of the transformed plant cells. These findings are in support by previous studies. Immunolocalization of host pathogenesis-related proteins between roots of Douglas-fir (DF; *Pseudotsuga menziesii*) seedlings and the laminated root rot fungus *Phellinus sulphurascens* showed that a thaumatin-like protein localized in host cell apoplast [45]. Using immunocytochemical method the authors showed that wheat TaPR5 protein was accumulated on cell walls of wheat leaves in the incompatible interaction [46]. These observations suggest that TLPs with acidic property and predicted signal peptide are secreted into apoplast of plant cell to function. While we have enough evidences to support the extracellular localization of TaLr35PR5 when it is transiently expressed in onion epidermal cells, it is still essential to further the investigation of the subcellular localization of TaLr35PR5 upon leaf rust pathogen infection. To achieve this purpose, either we can generate the transgenic wheat line carrying the fluorescent-tagged *TaLr35PR5* gene or develop the TaLr35PR5 antibody for the immunolocalization study in future experiment.

Although more than 20 TLPs from plant, animals and fungi have been reported to exhibit antifungal activity [47], specific mechanism underlying this function has not yet been completely elucidated. However, several antifungal actions have been described such as plasma membrane permeability via binding β-1,3-glucans of fungal cell wall [48, 49] and endo-β-1,3-glucanase activity for degradation of fungal cell wall [50]. Since TaLr35PR5 shares high homology with known PR5 family, such as TLPSec, possessing antifungal activity (Additional file 7: Figure S7), we speculate that it exhibits the same antifungal activity as well. However, it remains challenging to examine the antifungal activity in vitro as leaf rust is obligated pathogen and is not able be cultivated in vitro. To prove the antifungal activity of TaLr35PR5, we can consider injecting the purified protein to wheat leaves and monitoring the disease development after inoculating the rust pathogen. As an alternative approach, we can test the antifungal activity with purified protein against other fungal pathogens. But, this strategy will not provide the direct evidence on the anti-rust activity. Thus, examining the anti-rust activity of TaLr35PR5 with the fist approach will be the focus of our future study.

Our previous studies revealed that wheat *TaLr35PR5* is induced early and its expression level remains significantly higher in incompatible interaction compared with the compatible interaction. We therefore reasoned that TaLr35PR5 may be involved in *Lr35*-meidated resistance [34]. This turned out to be the case: when using BSMV-induced gene silencing approach, we observed that TcLr35 plants inoculated with *TaLr35PR5* silencing constructs exhibited more sever disease developments as evidenced by producing more leaf rust uredinia, which was scored as reaction type 3. While BSMV-induced gene silencing in barley and wheat has been becoming a popular tool for dissecting the wheat rust resistance genes-mediated signaling pathways in hexaploid wheat due to its high efficiency and rapid speed, it is still interesting to note that the BSMV-induced gene silencing efficiency remains variable depending on the target genes. BSMV-induced silencing of *RAR1*, *SGT1* and *HSP90* in wheat impaired *Lr21*-mediated resistance against leaf rust fungus, but the degree of suppression of *RAR1*, *SGT1* and *HSP90* were 54, 83 and 40%, respectively [51]. Knocking-down *TaNPSN11* (soluble N-ethylmaleimide-sensitive factor attachment protein receptors), *TaNPSN13*, and *TaSYP132* (plant defence-related *SNARE* homologues) expression using BSMV-induced gene silencing reduced the resistance of wheat to *Puccinia striiformis* f. sp. *tritici* (*Pst*) and the degree of suppression ranged from 70 to 99% [44]. Silencing three copies of the wheat (*Triticum aestivum*) *ADF* gene with efficiency higher than 65% resulted in the increased growth of *Pst* hypha compared with control plants upon inoculation with avirulent *Pst* [52]. In our study, we obtained 50 and 80% silencing efficiency using two different BSMV::V1 and BSMV::V2 silencing fragments (Fig. 5). Obviously, both silencing efficiency are largely different, which suggest that different silencing fragments that target the same gene can result in variable silencing efficiency. For future study, this has to be taken as a consideration in order to achieve optimal silencing efficiency. However, with the development and wide application of CRISPR/Cas9-based genome editing technology in wheat, it will hold the potential as a powerful tool to completely knockout an interesting gene in order to fully understand its biological function.

In our qPCR assay we showed that expression of *TaLr35PR5* in BSMV::V2 silenced plants is slightly higher than the expression in susceptible cultivar Thatcher (Fig. 5). However, we observed that the rust disease symptoms in BSMV::V2 silenced plant are much more less intensive compared to susceptible cultivar Thatcher (Fig. 4). There remains a discrepancy between the expression and disease severity. However, the similar expression of *TaLr35PR5* gene is insufficient to guarantee the similar accumulation of TaLr35PR5 protein. Since TaLr35PR5 protein is the functional factor involving in *Lr35*-meidated resistance, in the incompatible interaction accumulation of TaLr35PR protein could be higher than that in compatible interaction. To prove this hypothesis, detection of TaLr35PR5 accumulation is required for future study. An alternative explanation is that the function of TaLr35PR5 protein requires an interactor that is only present or activated after perception of corresponding avirulence protein by Lr35 protein. However, in the compatible interaction this interactor could be missing or inactivated. Recent findings showed that wheat pathogenesis-related protein 1 is targeted by necrotrophic pathogen ToxA and SnTox3, and *Botrytis cinerea* elicitor protein BcIEB1 interacts with the tobacco PR5-family to protect the fungus against its antifungal activity [53–55]. These findings broaden our understanding towards the potentially versatile function of PR proteins. Thus, we cannot rule out the possibility that TaLr35PR5 is able to interact with a wheat protein to form a functional complex. To prove this hypothesis, yeast two hybrid experiment is required to identify the interactor of TaLr35PR5 protein for future work.

## Conclusions

Taken together, our findings indicate TaLr35PR5 has a function in the defense response mediated by *Lr35* against leaf rust fungus. The information presented here underlines the importance of understanding the molecular function of wheat pathogenesis-related TLP in *R*-mediated resistance against leaf rust fungus. We demonstrate for the first time that silencing a pathogenesis-related thaumatin-like protein gene *TaLr35PR5* in wheat compromises *Lr35*-meidated resistance. These findings will expand our understanding towards the function of TLPs and will provide invaluable resources for understanding the molecular interaction between wheat and leaf rust pathogen.

## Additional files

Additional file 1: **Figure S1.** The amplified PCR product of signal peptide sequence was run on agarose gel. M:DL2000 marker; 1 and 2 are the individual amplified PCR product. One band with approximate size 100 bp is observed in lane 1 and 2. (TIF 129 kb)

Additional file 2: **Figure S2.** pSUC2::TaLr35PR5-SP digested with *XhoI* and *EcoRI* was run on agarose gel. M:DL2000 marker; 1 and 2 are the digested individual plasmid DNA. One band with approximate size 100 bp is present in lane 1 and 2, indicating that the insert is present in both the generated constructs. (TIF 205 kb)

Additional file 3: **Figure S3.** The amplified PCR product of full length TaLr35PR5 with *XbaI* and *KpnI* was run on agarose gel. M:DL2000 marker; 1 is the individual amplified PCR product. One band with approximate size 500 bp is observed in lane 1. (TIF 182 kb)

Additional file 4: **Figure S4.** pSUC2::TaLr35PR5-GFP digested with *XbaI* and *KpnI* was run on agarose gel. M:DL2000 marker; 1 and 2 are the digested individual plasmid DNA. One band with approximate size 500 bp is present in lane 1 and 2, indicating that the insert is present in both the generated constructs. (TIF 245 kb)

Additional file 5: **Figure S5.** The amplified PCR products of silenced fragment of TaLr35PR5 gene was run on agarose gel. M:DL2000 marker; 1 and 2 are the individual amplified PCR product. M: Marker 2000; 1. Amplified fragment of V1; 2. Amplified fragment of V2. (TIF 202 kb)

Additional file 6: **Figure S6.** Restrictive digestion of recombinant plasmid. M: Marker 2000; 1-2: Restrictive digestion of γ-V1, γ-V2 plasmid. (TIF 263 kb)

Additional file 7: **Figure S7.** Multiple sequence alignment of the predicted amino acid sequences of TaLr35PR5, TaPR5, PcOSM1, PcOSM2 and TLPsec. TaLr35PR5 shares 92% similarity with TaPR5 and TLPSec. The consensus amino acid sequences are highlighted. The 10 conserved cysteine residues are highlighted in dark red among the aligned proteins. The signal peptides are marked with rectangle. TaPR5 [46], Piper colubrinum [56], Sec, *Secale cereale* [57]. TLPsec, PcOSM1 and PcOSM2 with antifungal activity. (TIF 4064 kb)

## Abbreviations

AP: Apoplastic space
ER: Endoplasmic reticulum
PDS: Phytoene desaturase
PR: Pathogenesis-related
qRTPCR: Quantitative reverse transcription PCR
SAS: Systemic-acquired resistance
TLPs: Thaumamatin like proteins
YSST: Yeast signal sequence trap
