# Supplementary figures and images for: Functional analysis of a pathogenesis-related thaumatin-like protein gene TaLr35PR5 from wheat induced by leaf rust fungus
Source: BMC Plant Biol. 2018 May 4;18:76. doi: 10.1186/s12870-018-1297-2 (PMC5935958; doi:10.1186/s12870-018-1297-2)

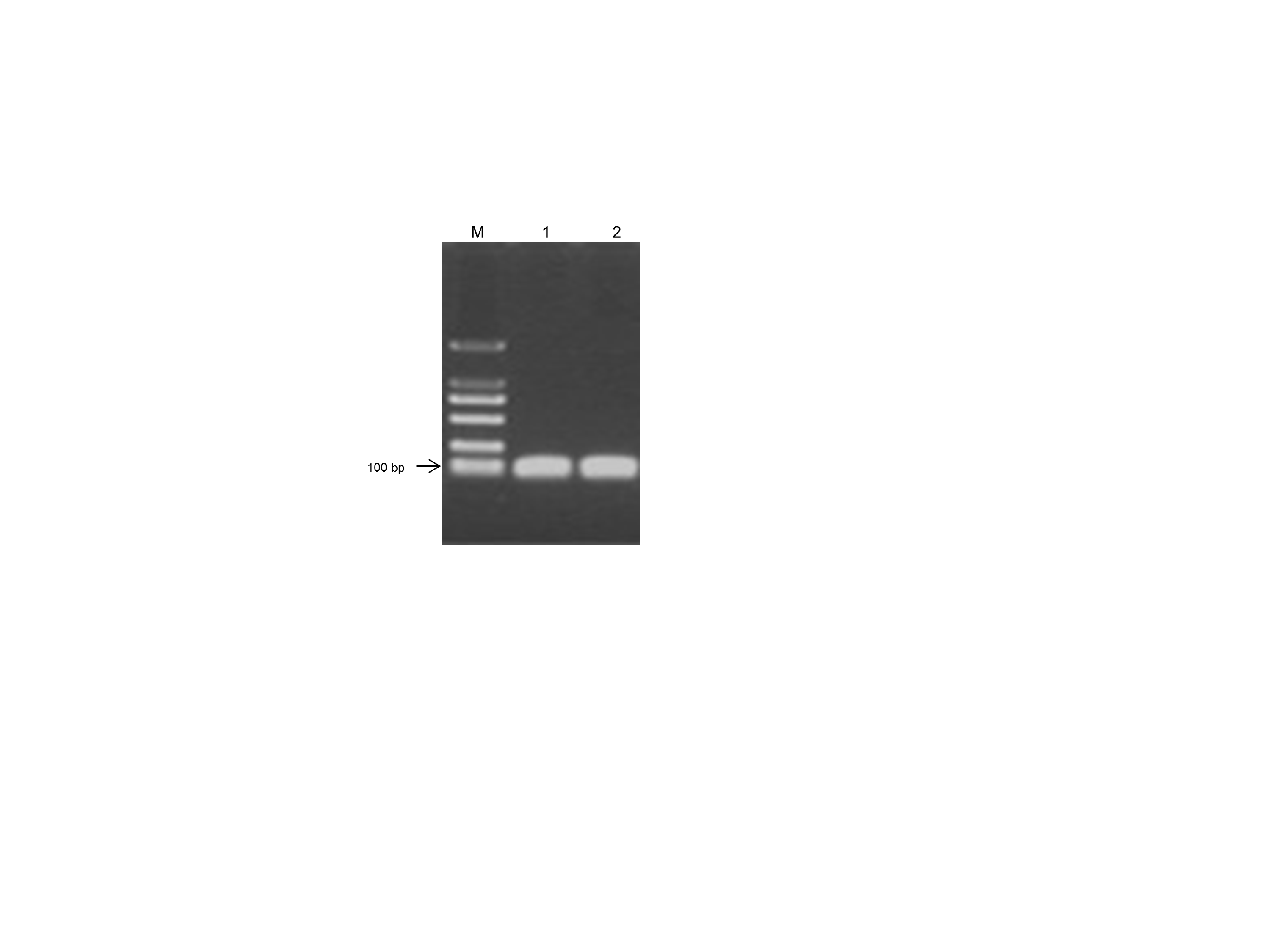

Supplement: Supplementary file 1 — Figure S1. The amplified PCR product of signal peptide sequence was run on agarose gel. M:DL2000 marker; 1 and 2 are the individual amplified PCR product. One band with approximate size 100 bp is observed in lane 1 and 2. (TIF 129 kb) [file 12870_2018_1297_MOESM1_ESM.tif]

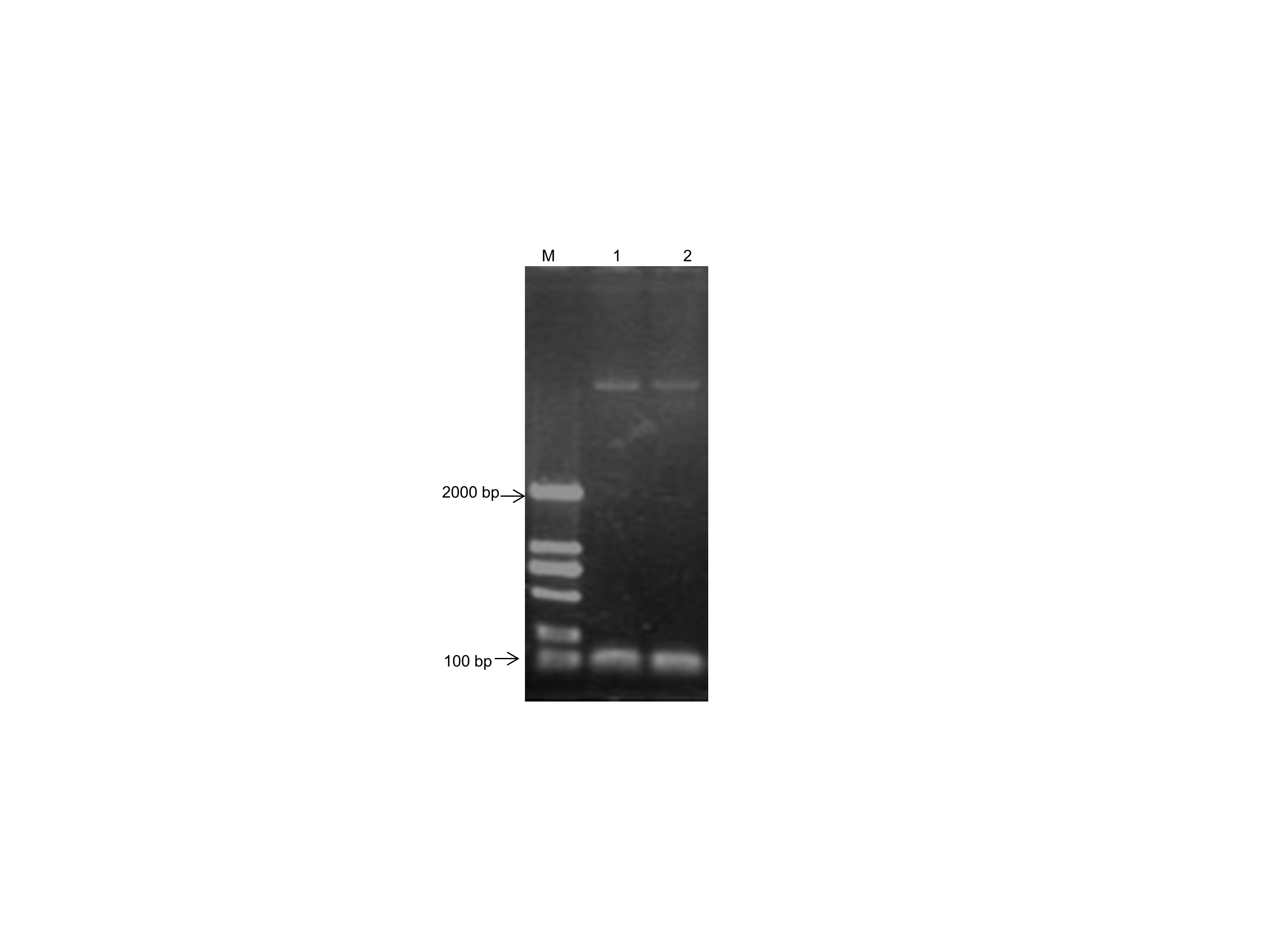

Supplement: Supplementary file 2 — Figure S2. pSUC2::TaLr35PR5-SP digested with XhoI and EcoRI was run on agarose gel. M:DL2000 marker; 1 and 2 are the digested individual plasmid DNA. One band with approximate size 100 bp is present in lane 1 and 2, indicating that the insert is present in both the generated constructs. (TIF 205 kb) [file 12870_2018_1297_MOESM2_ESM.tif]

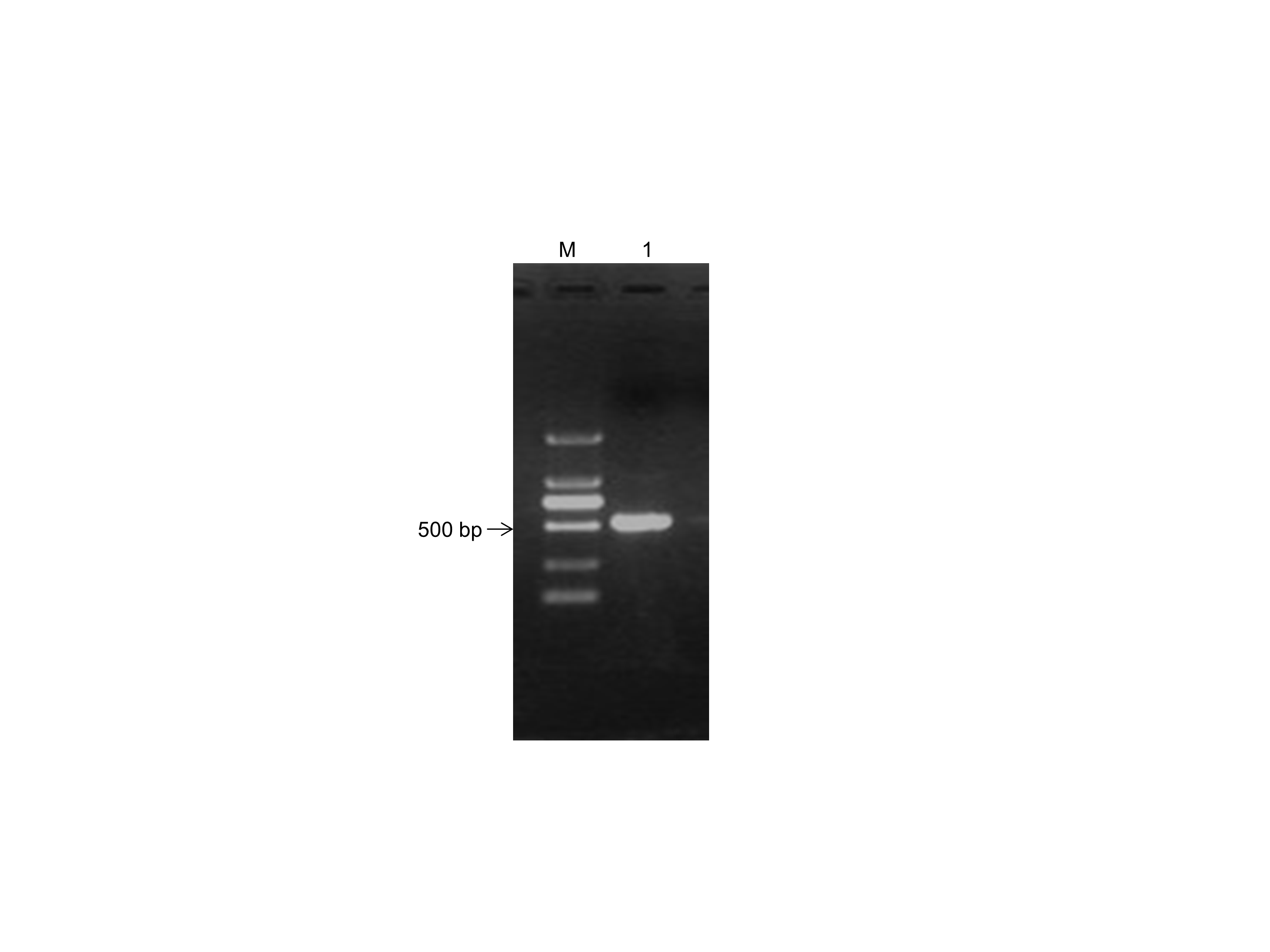

Supplement: Supplementary file 3 — Figure S3. The amplified PCR product of full length TaLr35PR5 with XbaI and KpnI was run on agarose gel. M:DL2000 marker; 1 is the individual amplified PCR product. One band with approximate size 500 bp is observed in lane 1. (TIF 182 kb) [file 12870_2018_1297_MOESM3_ESM.tif]

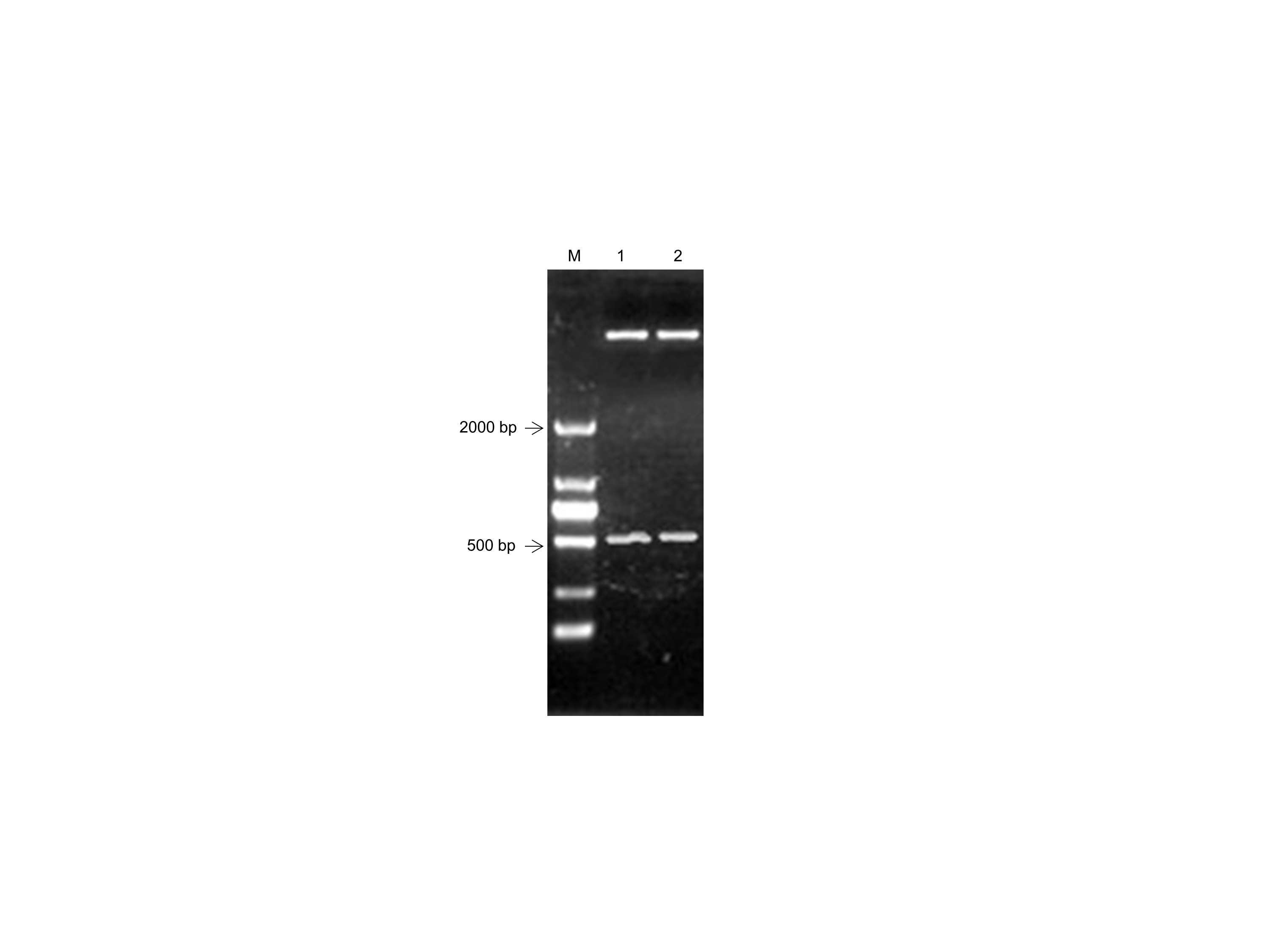

Supplement: Supplementary file 4 — Figure S4. pSUC2::TaLr35PR5-GFP digested with XbaI and KpnI was run on agarose gel. M:DL2000 marker; 1 and 2 are the digested individual plasmid DNA. One band with approximate size 500 bp is present in lane 1 and 2, indicating that the insert is present in both the generated constructs. (TIF 245 kb) [file 12870_2018_1297_MOESM4_ESM.tif]

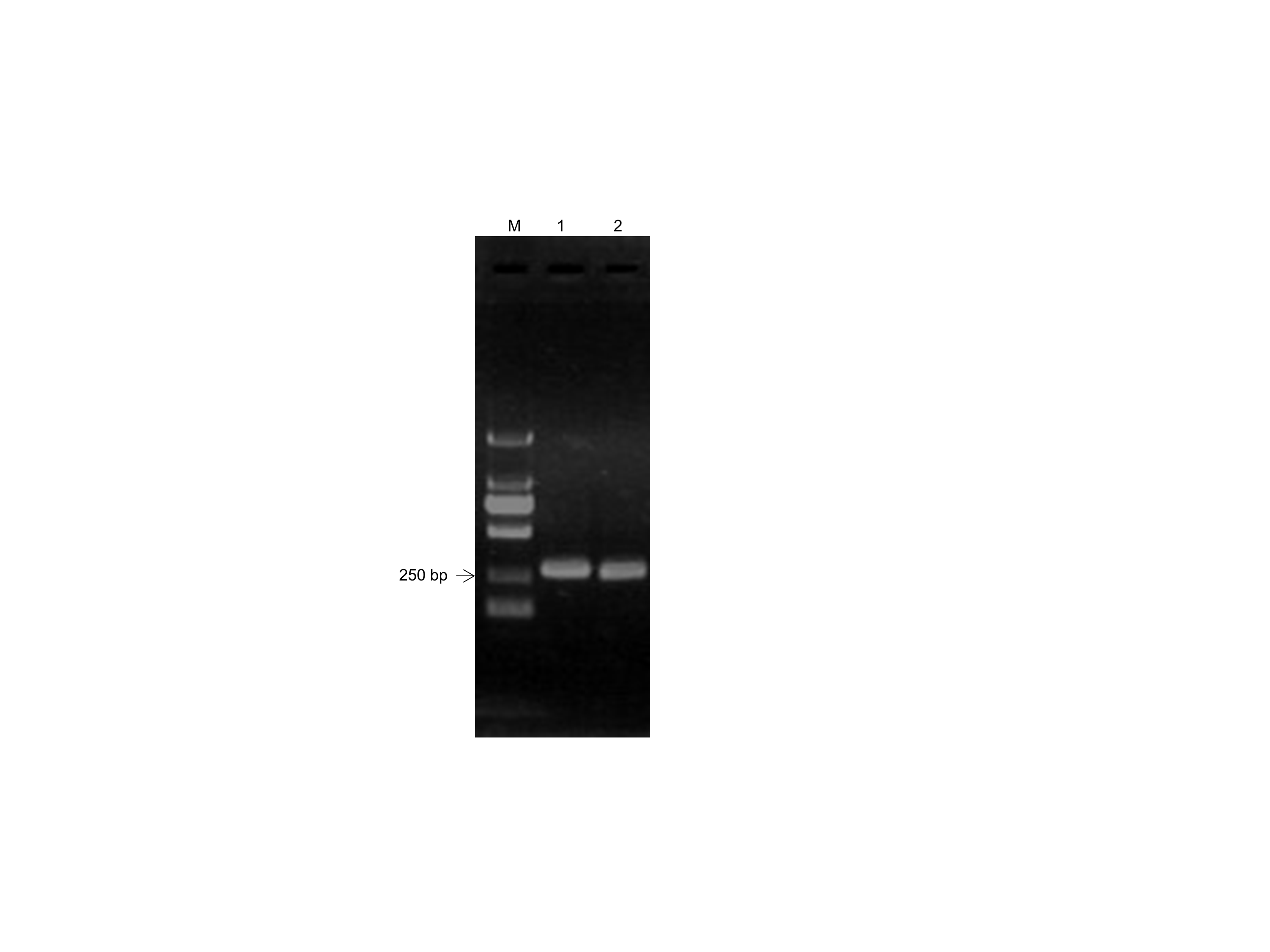

Supplement: Supplementary file 5 — Figure S5. The amplified PCR products of silenced fragment of TaLr35PR5 gene was run on agarose gel. M:DL2000 marker; 1 and 2 are the individual amplified PCR product. M: Marker 2000; 1. Amplified fragment of V1; 2. Amplified fragment of V2. (TIF 202 kb) [file 12870_2018_1297_MOESM5_ESM.tif]

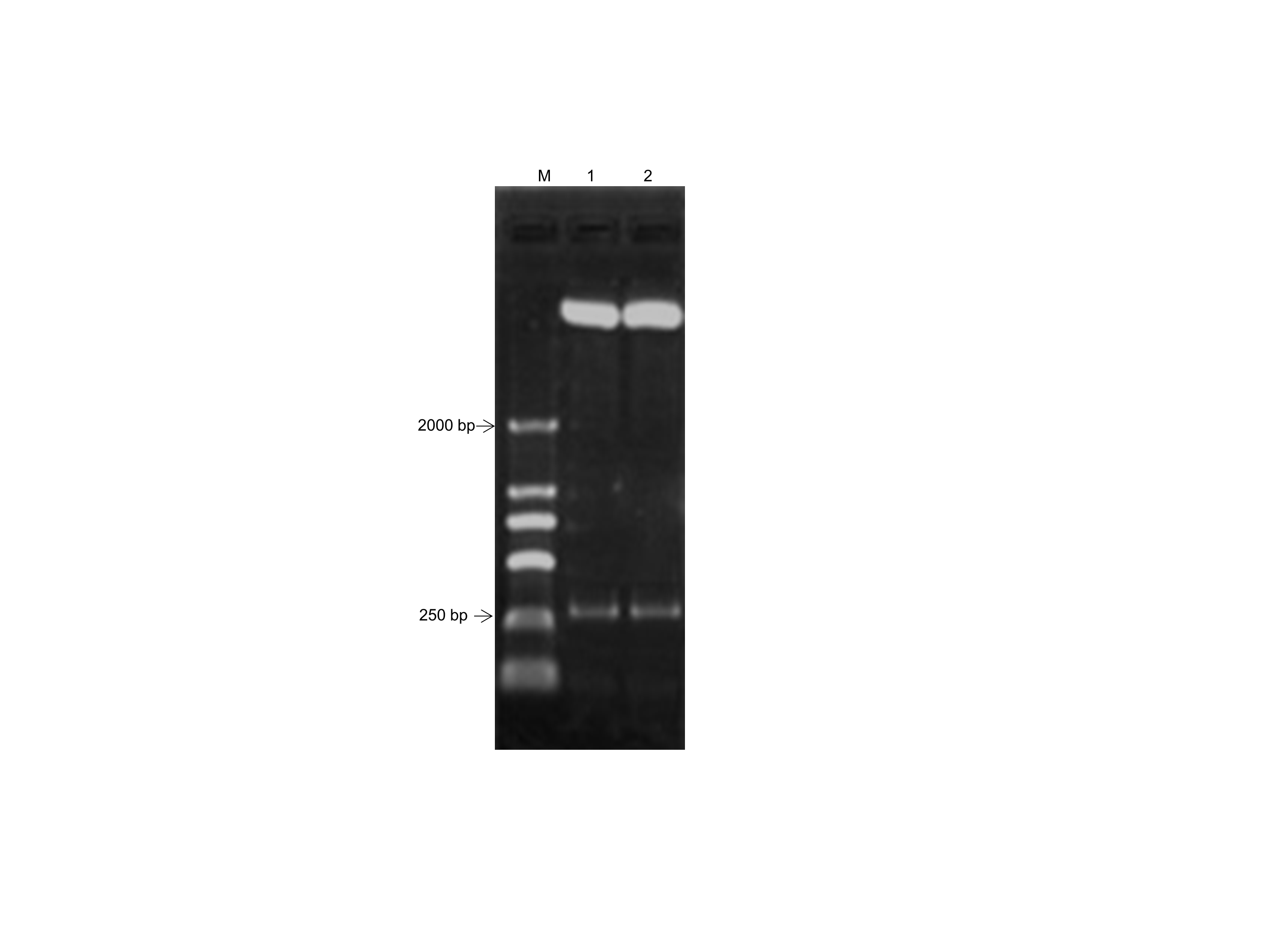

Supplement: Supplementary file 6 — Figure S6. Restrictive digestion of recombinant plasmid. M: Marker 2000; 1-2: Restrictive digestion of γ-V1, γ-V2 plasmid. (TIF 263 kb) [file 12870_2018_1297_MOESM6_ESM.tif]

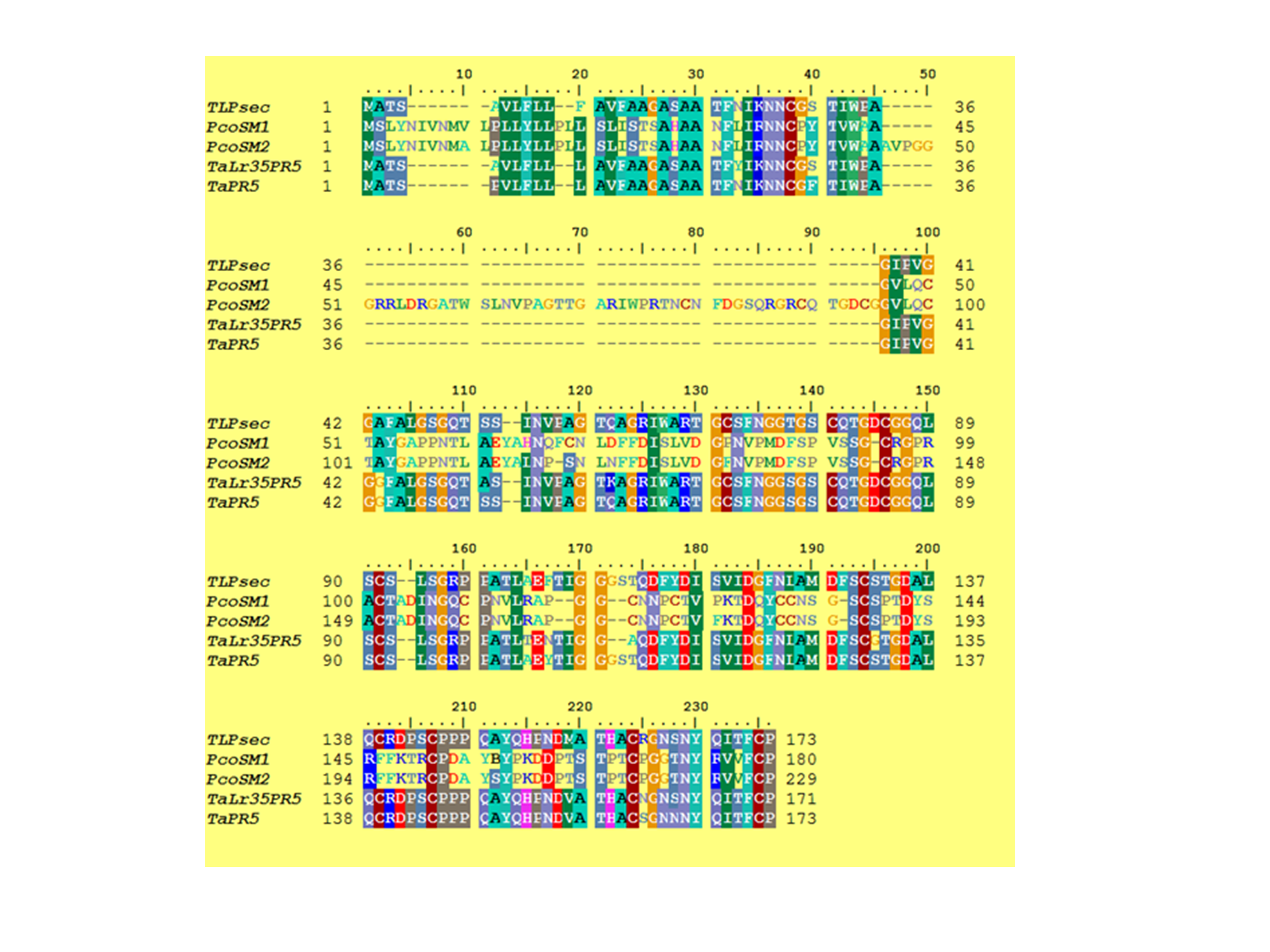

Supplement: Supplementary file 7 — Figure S7. Multiple sequence alignment of the predicted amino acid sequences of TaLr35PR5, TaPR5, PcOSM1, PcOSM2 and TLPsec. TaLr35PR5 shares 92% similarity with TaPR5 and TLPSec. The consensus amino acid sequences are highlighted. The 10 conserved cysteine residues are highlighted in dark red among the aligned proteins. The signal peptides are marked with rectangle. TaPR5 [46], Piper colubrinum [56], Sec, Secale cereale [57]. TLPsec, PcOSM1 and PcOSM2 with antifungal activity. (TIF 4064 kb) [file 12870_2018_1297_MOESM7_ESM.tif]
